# Mixed-Method Study of Utilizing Portfolios to Document and Assess Co-Curricular Activities: Student and Advisor Perceptions

**DOI:** 10.3390/pharmacy7040170

**Published:** 2019-12-13

**Authors:** Victoria Belousova, Amany K Hassan, Stacie Lampkin

**Affiliations:** 1 Department of Pharmaceutical, Social and Administrative Sciences, D’Youville School of Pharmacy, Buffalo, NY 14201, USA; hassana@dyc.edu; 2 Department of Pharmacy Practice, D’Youville School of Pharmacy, Buffalo, NY 14201, USA; thomans@dyc.edu

**Keywords:** co-curricular, portfolio, pharmacy education, affective domain, professional development

## Abstract

**Background**: Development of professional behaviors must occur in tandem with clinical skills to ensure graduates provide quality care. Portfolios have been widely utilized as a medium to document and reflect on experiences related to professional skills. **Methods**: Students were required to complete a series of co-curricular activities and document them via paper or electronic portfolios, which were shared with their advisors for feedback and review. To gather perception data, student surveys were administered twice: once for the electronic cohort and once for the paper cohort after their first-year experience with the platform, and focus groups were conducted a year later. Faculty advisors were also asked to complete surveys. **Results**: Both students and advisors felt that electronic portfolios resulted in a greater understanding of the educational outcomes and was the preferred method for recording co-curricular requirements. Several technical challenges arose with the use of the electronic portfolio and many students and advisors felt they needed more education regarding mapping of activities. **Conclusions**: The electronic portfolio was found to be more sustainable as compared with paper portfolios, as it helped students adhere to the criteria and self-assessment process. Further research is needed to evaluate long-term benefit of documenting and assessing co-curricular experiences within an electronic platform.

## 1. Introduction

The Accreditation Council for Pharmacy Education (ACPE) develops competencies, or outcomes, that all graduating student pharmacists must meet to succeed in the ever-changing world of healthcare. The standards released in 2016 emphasize the development of clinical skills in tandem with professional skills, aligning with the ongoing shift in the realm of pharmacy practice today [1]. Focusing on skills such as collaboration, leading by influence, entrepreneurialism, and self-awareness are vital to ensure that graduating pharmacists deliver quality patient-centered care [2]. 

While a majority of the 2016 ACPE accreditation standards can be introduced and assessed within the parameters of the curriculum, professional behaviors are not passive and therefore cannot be imparted through simple lecture—they must be acquired through active participation in activities that foster their growth [3]. Co-curricular activities are learning experiences that complement and advance the learning that occurs within the formal didactic and experiential curriculum [4] and are a crucial part of the professional development of student pharmacists. Engaging in co-curricular activities allows students the opportunity to apply concepts introduced within the classroom in real-world scenarios, and fit best for the development of professional skills and behaviors. 

In early 2000, the White Paper on Pharmacy Student Professionalism emphasized the need and benefit of documenting these professional skills in portfolios to serve as, “…a constant reminder of the commitment [students] have made to society as a professional and the progress they have made along the lifelong process of becoming one” [3]. More importantly, portfolios have been shown to allow for the integration and linking of theory into practice [5]; just because a student is introduced to the concept of leadership, does not mean that they have exceptional leadership abilities. Documenting and reflecting on leadership-related co-curricular activities via a portfolio provides an opportunity for the student to self-assess their skills and identify areas for improvement. It can therefore be inferred that documentation of co-curricular activities via a portfolio is vital to the continued professional development of student pharmacists. 

Portfolios have been utilized as an effective tool to collect, showcase, and assess a student’s accomplishments across a variety of disciplines and can be either reflective or demonstrative [6,7,8]. While demonstrative portfolios allow a student to showcase their best work, reflective portfolios provide the student with the best opportunity to show development within an area and are of greater use in helping students achieve the educational outcomes listed in Standard 3 and 4 of the 2016 ACPE standards, which are associated with professional skills and behaviors [9]. 

There are two main types of portfolio platforms that can be utilized for reflection—paper and electronic (ePortfolio). Due to the complexity of documenting and assessing portfolios, some may be more comfortable with a paper system, but an electronic portfolio allows for better cumulative assessment required for ACPE to meet standards. Based on recent estimates, roughly 88% of pharmacy schools across the United States utilize a portfolio within their curriculum [9], but a current best practice does not exist. Furthermore, while students often regard portfolios as important tools to enhance their learning process and allow them to reflect on their abilities, [9,10] the question arises as to whether they perceive a greater benefit from one versus the other. 

The objective of this study was to describe the development process of a newly implemented co-curricular framework and to evaluate student and advisor perceptions of the benefits of utilizing two different portfolio platforms to document evidence and reflect on the development of professional skills and behaviors.

## 2. Materials and Methods 

### 2.1. Co-Curricular Framework: Development, Overview, and Integration with Curriculum

The 2016 ACPE standards lists four broad categories of educational outcomes that graduates must achieve prior to matriculation: foundational knowledge (Standard 1), essentials for practice and care (Standard 2), approach to practice and care (Standard 3), and personal and professional development (Standard 4) [4]. Assessment of these outcomes is achieved through a combination of curricular assessment through examinations and co-curricular assessment via the portfolio system.

Eight educational outcomes from Standard 3 and 4 were identified as being associated with professional skills and behaviors and therefore considered to be co-curricular: education (3.2), interprofessional collaboration (3.4), cultural sensitivity (3.5), self-awareness (4.1), leadership (4.2), innovation and entrepreneurship (4.3), professionalism (4.4), and service/civic engagement (4.4.2). These were chosen as they are not easily documented in the standard curriculum and reflect most of the work that students performed outside of the classroom.

Students were required to provide evidence of completing at least one activity from each of the eight co-curricular educational domains by the end of their third professional year to demonstrate achievement. Refer to Appendix A for more information. 

Activities that were already part of a didactic or experiential course were not eligible for this requirement: If a student was mandated to attend a guest lecture for one of their courses, they would not be able to use this as a co-curricular activity. A list of sample activities associated with each of the outcomes can be found in Table 1. 

In the fall of 2016, the class of 2020 started developing their paper-based portfolios and utilized documentation forms to record their learning experiences. The following year, in the fall of 2017, a student-driven ePortfolio was developed in conjunction with PharmAcademic, a platform developed by the McCreadie Group, Inc. that allows schools to manage and track elements related to education. This allowed students in the class of 2021 to record their co-curricular experiences in an electronic format. 

Both portfolios were incorporated into courses as a completion grade; successful completion of their portfolios was a requirement for passing the course.

Students from both cohorts followed similar submission procedures after participating in activities; documentation consisted of self-assessment using reflective writing and submission of verifiable artifacts, such as certificates of completion. Additionally, students mapped each activity to one of the eight co-curricular educational outcomes to demonstrate completion. Students in the class of 2021 also mapped each activity to Bloom’s taxonomy. Both cohorts participated in workshops aimed at explaining the purpose, components, and assessment of the co-curriculum, including a session on writing reflections and mapping to educational outcomes. Furthermore, students in the ePortfolio cohort received additional training on the use of PharmAcademic to document activities.

The self-directed learning model was used throughout: students utilized reflections as a self-assessment tool within the portfolio to evaluate progression in and achievement of the educational outcomes. To ensure sustainability, an advisor-driven process was developed wherein advisors reviewed student submissions and reflections to ensure they were aligned with goals and outcomes. Advisors were also encouraged to utilize student activities as talking points to encourage the growth of students. Furthermore, advisors were responsible for providing a final sign-off at the end of each semester to ensure that their advisee completed the required activities and demonstrated successful achievement of the educational outcomes associated with them.

To ensure success, several educational sessions were offered to faculty advisors to improve their knowledge of the self-directed learning model, student requirements, the educational outcomes, and how to improve communication with their advisees. Reflection-writing guidelines were also shared with advisors to standardize the evaluation process. In addition, advisors participated in sessions explaining the process of reviewing evidence within the ePortfolio.

### 2.2. Data Collection

The study was approved by the Institutional Review Board at D’Youville. Data for the study was collected via cross-sectional surveys and focus groups. All participants gave their informed consent for inclusion before they participated in the study. 

Students in the class of 2021 and class of 2020 were asked to complete a survey administered via Qualtrics^®^ after their first-year experience with their respective platforms, as these were the only two cohorts completing the portfolio process at the time. The survey included four sections: demographics, perceived benefits of documenting evidence, perceived benefit of the portfolio, and ease of use. Items about the perceived benefit of the portfolio and ease of use of the electronic platform were rated using a 4-point Likert scale (strongly agree, agree, disagree, and strongly disagree). Students were also given the opportunity to comment on their challenges and ideas for process improvement. Survey questions were developed from study objectives.

Faculty advisors were asked to complete a survey administered via Qualtrics^®^ regarding their experiences with the portfolio process at the same time as the students. The survey had five sections to mimic the student survey: demographics, perceived benefits of learning, perceived value, ease of use, and ePortfolio vs. paper comparison. Advisors were also given the opportunity to comment on their challenges and ideas for process improvement. 

Though not pre-planned, focus groups were added to the project to gain a better understanding of common themes described in the comment sections of student surveys. A total of 17 open-ended questions were developed (14 generalized questions and 3 related to the ePortfolio) based on responses to the survey: for example, if responses indicated lack of support from advisors, students were asked “Can you describe a typical meeting with your advisor where you discuss the co-curricular framework? How can your advisor help you, from your perspective, in relation to the co-curricular framework and helping you track your progress?” All focus group questions can be found in Appendix B. 

Ten students were randomly selected from each cohort during the spring semester of the second year of portfolio use. Selected students were offered a $25 gift card for their participation; if a student declined to participate, another student was randomly selected. The focus groups themselves were conducted as semi-structured interviews lasting 60-mins each, with each cohort being split into two groups of five students. Facilitators were administrators or fourth year students to avoid social desirability bias from students. The authors met with the groups’ facilitators to explain the purpose of the study and review the questions and prompts of each of the questions. 

### 2.3. Data Analysis

Results from the surveys were summarized using means and standard deviation for continuous variables or frequencies and percentages for categorical variables. Chi square and Fisher’s exact tests were used to examine the difference in response to some survey items between cohorts. Survey analysis was conducted using SPSS V 25.0 (IBM Corp. Released 2017. IBM SPSS Statistics for Windows, Version 25.0. Armonk, NY: IBM Corp) using significance level of 0.05.

Each focus group interview was transcribed by work-study students (NB, HAN) and independently coded by a study investigator (AH) and a work-study student (HAN). Themes were not predetermined and instead were identified after review. Quotes that most accurately summarized the themes and subthemes were also identified. 

## 3. Results 

### 3.1. Student Survey Results 

We received 62 responses (93.9%) from the class of 2020 and 41 responses (93.2%) from class of 2021. There were no differences in gender distribution between the two classes, with 62.9% and 58.5% being females for the classes of 2020 and 2021, respectively (*p* = 0.884). There was also no difference in the perceived competency in information technology, with 75.8% rating their skills as average in the 2020 class compared to 73.2% in the 2021 class (*p* = 0.655).

Table 2 shows the number of students who agreed with the survey items, broken down by class year. In comparison to the class of 2020, the class of 2021 had a higher, statistically significant, percentage of students agreeing the portfolio process improved their understanding of the educational outcomes (100% [ePortfolio] vs. 67.7% [paper], *p* < 0.0001) and that it was valuable (80.5% [ePortfolio] vs. 56.6% [paper], *p* = 0.012). Furthermore, there was a significant difference in general attitude towards the co-curricular framework between the two groups: 73.2% positive (ePortfolio) vs. 46.8% positive (paper), *p* = 0.008. 

Generally, students preferred recording their activities in an ePortfolio as compared to the paper portfolio (70.7% [ePortfolio] vs. 29.0% [paper], *p* < 0.0001) and did not have any difficulties retrieving their evidence in the ePortfolio as compared with the paper portfolio (100% [ePortfolio] vs. 72.6% [paper], *p* < 0.0001). Additionally, most students agreed that the ePortfolio was a convenient place for them to store their activities (92.7%). Although not the majority, a high percentage of students agreed that they had difficulties keeping track of their experiences in their designated formats (41.5% [ePortfolio] vs. 48.4% [paper], *p* = 0.490).

### 3.2. Student Focus Group Results

A total of 10 students from the class of 2020 and 9 students from the class of 2021 participated in the focus groups. Data coding showed that themes from both groups of each cohort were almost identical, so no further focus groups were scheduled. Overall, five major themes were identified for each cohort: Paper: (1) co-curricular requirements are major factors in the decision to participate in activities, (2) increased burden and loss of autonomy, (3) generally viewed as valuable but time consuming, (4) need to train students, (5) need to train advisors; ePortfolio: (1) co-curricular requirements are major factors in decision to participate followed by time constraints, (2) co-curricular activities and portfolio documentation viewed as valuable to their professional development, (3) there is a need for further training to improve mapping and use of the ePortfolio, (4) mostly the use of the ePortfolio to track activities and progression was favored by students, (5) there is a need to educate students about the purpose of co-curricular requirements and the self-assessment process and encouraging more autonomy. Overall, there were several similarities in themes: the need for more training in the general process and the educational outcomes; students selecting activities based on requirements instead of those relevant to their interests; students would have preferred a pre-mapped list of activities to choose from to make the mapping process easier. Common themes along with response summaries and quotes can be found in Table 3 and Table 4.

### 3.3. Faculty Advisor Survey Results

A total of 23 faculty advisors were asked to complete the survey and a total of 13 responses were received. Advisors were predominantly female (69%) and on average had 2 advisees per semester. Table 5 shows the number of advisors who agreed or strongly agreed with the survey items. 

Of those advisors who responded to the survey, 92% (12/13) agreed that creating and maintaining portfolios made students more aware of the educational outcomes. When asked about general attitudes regarding the paper and electronic portfolios, results tended to be overall positive (66% paper and 69% electronic). Despite this, 84% (11/13) of advisors stated that they preferred utilizing the ePortfolio for evaluating advisee progression, as it was easier to keep track of activities in an electronic format. As one advisor stated in the comment section:

“Any method of streamlining files and information to an electronic format that is all housed in one place is preferable. While the ePortfolio hasn’t been without issues, it is a step in the right direction and I think it is a necessary transition to move towards an electronic format.”

When asked for feedback regarding improvements, most advisors wanted a uniform framework with additional training sessions.

## 4. Discussion

Overall the results of the study showed that students perceived the new co-curricular requirements to be beneficial to their professional development as future pharmacists. This is similar to results by Hoffman et al., where the authors concluded that students perceived co-curricular activities to supplement the didactic portion of the pharmacy curriculum and promoted professional development outside of a traditional classroom setting [11]. Regardless of which platform was utilized, the students found the required co-curricular activities and reflection process valuable for improving their understanding of the educational outcomes, pushing them outside of their comfort zones to try new activities, providing opportunities for students to reflect on the reasons behind activities, and encouraging them to reflect on personal strengths, weaknesses, and professional goals. Advisor results are also reflective of this trend: 92% agreed that it made students more aware of the educational outcomes and 85% agreed they provide places for students to reflect on strengths and weaknesses. 

Students’ comments indicated that there was a lack of understanding of the purpose of reflection as a self-assessment tool and not simply done to fulfill course requirements. In addition, several comments from the focus groups revealed concerns about loss of autonomy by having a mandate of educational outcomes that may not be aligned with the students’ interests or career goals. In other words, students participated in activities to fill requirements instead of focusing on which activities were likely to facilitate growth. This was despite the use of general categories from the eight educational outcomes to give students the freedom to select activities that best suit their interest. Additional training sessions were scheduled throughout the curriculum for students to gain a deeper understanding of the purpose of reflective writing. 

Another concern that was identified from the study results was confusion among advisors. The process at D’Youville School of Pharmacy started in 2016 with the introduction of the paper portfolio, which transitioned into the ePortfolio in the fall of 2017. The decision was made to allow the paper cohort to continue with their platform rather than transitioning them to the ePortfolio. As such, there were two sets of requirements and guidelines, leaving some advisors feeling ill prepared and not comfortable with the new format. This is evident of students in the ePortfolio cohort feeling like they were not as supported by their advisors (100% vs. 90.2%, *p* = 0.012); focus group results provided further evidence that many found their advisors just as confused about the process as students were, which translated to a feeling of lacking support.

These results were similar to the study conducted by Lucas et al., which compared pre/post use data of ePortfolio use in student pharmacists [12]. The authors noticed a significant difference between pre- and post-use student survey results, specifically that post-use survey results yielded lower means than pre-survey results within most area. Lucas et al. concluded that this was dependent on the structure and organization of the portfolio process, something that we experienced first-hand. 

One major area that students’ comments indicated the need for additional training in was on the topic of mapping, or relating a completed activity to an educational outcome. During focus groups, many students mentioned that they would rather have a list of available activities pre-mapped to an educational outcome rather than having to map it themselves. While this may be a viable solution, this does not encourage students to reflect on the reasons behind the activities that they are doing, which is the whole purpose of the process. Furthermore, variability exists between student experiences as it relates to the educational outcomes: two students may participate in a blood pressure clinic, but one may map it to educator while the other maps it to cultural sensitivity. These results emphasized the importance of an ongoing training process and having a buy-in from both students and advisors.

The study results showed multiple benefits when it came to the use of the ePortfolio for documenting co-curricular activities. First, as with any electronic process, it was more convenient and required less paperwork and students had to follow a specific format for documentation, and, ensuring that evidence was mapped appropriately, outcome reports were accessible, providing easier summation of educational outcomes for assessment purposes. Next, because the paper platform heavily relied on students and advisors manually keeping track of due dates, requirements, and final signoffs, there were instances where some deadlines were missed. In contrast, the ePortfolio provided automatic prompts and reminders for assignments, due dates, and signoffs for both students and advisors. 

Lee et al. reported that 62% of students found the ePortfolio helped them become more effective and independent learners. They also found that 65% of students agreed that the ePortfolio allowed them to reflect on areas of weakness [13]. Ashcroft and Hall found that 60% of students felt they gained insight into their learning and 48% of students stated they used paper portfolios as a place to identify strengths and weaknesses [14]. Our results were on par with this data: 67.7% and 100% for gaining insight into learning and 53.6% and 46.4% for identification of strengths and weaknesses for the paper and ePortfolio, respectively. These results indicate that portfolios were not only beneficial for being utilized for storage of artifacts, but also demonstrate the strength of the self-directed learning model. 

While the use of ePortfolios helped streamline the process, some challenges still arose. One of the major downsides of the paper portfolio is manual tracking of material and theoretically, ePortfolios should have removed this burden, however we found that there was no difference between the two cohorts in terms of these difficulties (48.4% vs. 41.5%, *p* = 0.490), and many students noted technical difficulties throughout the process. Similar to other published literature [12], we had technological challenges, but this is expected with any electronic medium, however, because PharmAcademic was altering their software and developing new tools to be able to meet our needs, there were some errors and system outages, leading to some frustration on behalf of students and advisors. Despite this, the general attitude for the ePortfolio remained significantly more positive among students compared to the paper portfolio, indicating that the quality improvement processes that were implemented lead to favorable results.

The study has several limitations. First, there is no control group; we had to use two different student cohorts which may have resulted in bias from the 2020 class since the initial implementation also included a learning curve for both students and advisors. However, this also applies for the initial roll out of the ePortfolio through PharmAcademic, which included similar challenges. We did not use a rubric to assess the students’ reflections; students were required to answer three core questions in their reflections, but there might have some variability between students with regards to what the reflection means or what is required. Moreover, the results of the surveys and focus groups were cross-sectional after the first year of implementation of each platform, not longitudinal. We did use the results from the survey to inform the discussions of the focus groups which took place almost a year after the administration of the survey, but they were not meant to measure a change in perceptions over time. The perceptions of the students may have changed after getting familiar with the process or platform, but this was not captured in our results. Additionally, due to the sample size it would be difficult to generalize about the utilization of this platform and process for institutions of a larger size. Further research is needed to evaluate long-term benefit of documenting and assessing co-curricular experiences within an electronic platform. 

## 5. Conclusions

Our program implementation of co-curricular requirements and the assessment process overall was perceived positively by the students. However, there were multiple challenges with using both a paper-based versus an electronic portfolio. The use of an ePortfolio is more sustainable and helped students adhere to the criteria and the self-assessment process. 

## Figures and Tables

**Table 1:** Examples of co-curricular activities and corresponding educational outcomes.

Outcome	Description	Sample Activity
3.2: Education	Educate all audiences by determining the most effective and enduring ways to impart information and assess learning.	Conduct “brown bag” medication reviews. Participate in drug abuse/unintentional misuse (poisonings) education programs.
3.4.1: Collaboration	Effectively collaborates with health care professionals, policymakers, administrative, and support personnel to engender a team approach to patient-centered care.	Join in advocacy initiatives (letter writing campaign). Participate in professional or interprofessional associations.
3.5: Cultural Sensitivity	Recognize social determinants of health to diminish disparities and inequalities to access to quality care.	Partake in seminars or initiatives that address global health disparities. Volunteer at a pro-bono clinic for underserved/impoverished citizens.
4.1: Self-Awareness	Examine and reflect on personal knowledge, skills, abilities, beliefs, biases, motivation, and emotions that could enhance or limit personal and professional growth.	Participate in professional development workshops (curriculum vitae preparation, interviewing skills, residency preparation).
4.2: Leadership	Demonstrate responsibility for creating and achieving shared goals, regardless of position.	Participate in local, state or national pharmacy, or scientific organization meetings. Serve on school or college committees.
4.3: Innovation and Entrepreneurship	Engage in innovative activities by using creative thinking to envision better ways of accomplishing professional goals.	Create/initiate a plan, protocol, event, or program to improve current practices related to the pharmacy profession.
4.4: Professionalism	Exhibit behaviors and values that are consistent with the trust given to the profession by patients, other healthcare providers, and society.	Participate in local, state, or national competitions that focus on patient counseling or clinical skills. Participate in legislative day events and advocacy initiatives.
4.4.2: Service (Civic Engagement)	Demonstrate compassion, productivity, and responsibility by serving in volunteer community activities.	Participate in walks, rides, runs, benefits, or other activities that fundraise for or directly donate goods to charitable organizations.

**Table 2:** Total number of students strongly agreeing or agreeing to survey items.

Survey Item	Class of 2020 Paper N = 62, (%)	Class of 2021 Electronic N = 41, (%)	*p* Value
Improved understanding of the educational outcomes.	42 (67.7)	41 (100.0)	<0.0001
Encouraged me to think about the reasons behind activities.	39 (62.9)	37 (90.2)	0.002
Made me a more effective learner.	31 (50.0)	28 (68.3)	0.066
Given me the opportunity to fine tune my organizational skills.	42 (67.7)	31 (75.6)	0.390
Encouraged me to reflect on personal strengths, weaknesses, and professional goals.	37 (53.6)	32 (46.4)	0.052
Determine the steps I need to take to achieve my professional goals.	35 (56.6)	30 (73.2)	0.085
Making a portfolio was a valuable activity.	35 (56.6)	33 (80.5)	0.012
I would have participated in all activities even if it was not a requirement.	58 (93.5)	38 (92.7)	0.864
Had difficulties keeping track of my experiences and reflections.	30 (48.4)	17 (41.5)	0.490
Liked recording my experiences and reflections in the format.	18 (29.0)	29 (70.7)	<0.0001
Did not have difficulty retrieving evidence of my experiences and sharing them with my advisor.	45 (72.6)	41 (100)	<0.0001
Communicating with and updating my advisor about my experiences was a seamless process.	48 (77.4)	35 (85.4)	0.318
Felt supported by my faculty advisor.	62 (100)	37 (90.2)	0.012
Did not have difficulties categorizing by educational outcome.	62 (100)	29 (70.7)	<0.0001
Felt supported by the course coordinators.	56 (90.3)	38 (92.7)	0.678
Instructions for completing assignments and deadline were useful and readily available.	50 (80.6)	34 (82.9)	0.770
The professional development course helped me succeed.	41 (66.1)	37 (90.2)	0.005
Number of assignments were reasonable.	48 (77.4)	35 (85.4)	0.318
Time needed to complete assignments was reasonable.	58 (93.5)	37 (90.2)	0.540
I will share this with my future employer ^a^.	--	34 (82.9)	--
The ePortfolio was a convenient place to store my activities ^a^.	--	38 (92.7)	--

^a^ These questions were only asked of the ePortfolio cohort.

**Table 3:** Common themes in focus group comments for the paper portfolio (class of 2020).

	Theme 1	Theme 2	Theme 3	Theme 4	Theme 5
	Co-curricular requirements are major factors in decision to participate in activities.	Increased burden, loss of autonomy.	Generally viewed as a valuable activity but time consuming.	There is a need to train students.	There is a need to train advisors.
Summary of Responses	Most students agreed that they choose what they needed to fulfill the requirements.	Students agreed that there is a higher expectation now of what they need to do before they graduate. There was a concern that they cannot participate in activities of interest to them because of time constraints.	Students agreed that it pushed them out of their comfort zone to try new activities and the reflections made them think about what they do.	Difficulty mapping Several comments about wanting the school to provide a list of co-curricular activities ahead of time.	Lack of consistency among advisors.
Student Quotes	“I chose what was needed to fill requirement rather than what I would have liked to take.”	“More expected out of the students to prove that we are more wholesome as pharmacists.” “Better if there wasn’t a cap on what you have to do, more room to choose fewer but cooler ones.”	“Does make you have to step out of boundaries.” “Different experiences that you wouldn’t do on your own, things you might not feel comfortable doing, takes you out of your comfort zone.”	“Mapping is the hardest part, typically bounces ideas off other students.” “Evidence part is very confusing.” “Pictures for evidence.” “Give a list of activities where the students can choose from.”	“Students take it as one thing, and advisor takes something else.”

**Table 4:** Common themes in focus group comments for the ePortfolio (class of 2021).

	Theme 1	Theme 2	Theme 3	Theme 4	Theme 5
	Co-curricular requirements major factor in decision to participate followed by time-constraints.	Co-curricular activities and portfolio documentation viewed as valuable to their professional development.	There is a need for further training to improve mapping and use of e-portfolio.	Mostly the use of e-portfolio to track activities and progression was favored by students.	Need to educate students about the purpose of co-curricular requirements and the self-assessment process and encourage more autonomy.
Summary of Responses	Several responses indicated that students participated in some events just to meet the school requirements and had to forego other activities that they liked because the educational outcomes were already satisfied by activities they had already completed. Other factors included level of interest in topic and scheduling.	All students felt they understood the purpose of completing co-curricular activities. Most students thought that the process was useful and a valuable activity. Some felt the ePortfolio was necessary for documentation and that they would not have kept track of their progress otherwise.	There was confusion among several students about mapping to educational outcomes and made uploading evidence into the e-portfolio difficult. They had to go to the slides outlining the process for help or had to seek help from advisors.	Four out of 5 students thought the use of e-portfolio to tracking activities was easy.	Some students confused the evidence of learning with evidence of participation. Comments included use of a sign off sheet or pictures could replace the reflection. There were also comments that they preferred to choose activity based on interest alone, which defeats the purpose of exposing student pharmacists to different experiences to promote their professional development.
Student Quotes	“I wouldn’t do activities if they were not required.” “Different categories make you try different things outside of your normal interests.”	“Yes to ePortfolio being a valuable activity—I see what I learned and strengths and weaknesses.” “EPortfolio very useful for keeping CV up to date.”	“Uploading is the most confusing part.” “Figuring out what event fits what category. Can fix it by telling us this event is good for this category.”	“No [to difficulty in tracking activities] …all listed by what you did by date.”	“Idea of ePortfolio is good but not organized. Rather do things interesting to me instead of different outcomes and mapping.”

**Table 5:** Total number of advisors strongly agreeing or agreeing to survey items.

Survey Item	Responses N = 13 (%)
Creating and maintaining an ePortfolio has made my advisees cognizant about the educational outcomes.	12 (92.3)
Creating and maintaining an ePortfolio has made my advisees more effective and independent learners.	9 (69.2)
Creating and maintaining an ePortfolio has made my advisees think about the skills and knowledge they want to acquire and steps needed to achieve them.	10 (76.9)
The ePortfolio allows my advisees to reflect on their strengths, weaknesses, and identify areas for improvement.	11 (84.6)
Creating and maintaining the ePortfolio was a valuable tool for my advisees in helping prepare them for a career of lifelong learning.	12 (92.3)
Creating and maintaining my ePortfolio was a valuable activity for my advisees.	11 (84.6)
It was easy for my students to keep track of their experiences via the ePortfolio.	8 (61.5)
The ePortfolio was a convenient place for my advisees to store examples of their work.	10 (76.9)
It was easy and convenient for me to review the experiences and reflections of my students via the ePortfolio.	8 (61.5)
Communicating with and being updated by my advisee about their experiences was seamless.	5 (38.4)
The instructions provided to me, as an advisor, were a useful resource.	11 (84.6)
The instructions provided to my advisees were a useful resource.	11 (84.6)
The instructions for deadlines and completing assignments were readily available and useful.	11 (84.6)
The number of assignments per semester/year were reasonable.	13 (100.0)
The amount of time needed to complete assignments was reasonable.	12 (92.3)
It was easy and convenient for me to review the experiences and reflections of my students in the class of 2020 through their paper portfolios.	7 (53.8)
Keeping track of activities in a paper format was a valuable activity for my advisees.	7 (53.8)

## Appendix A. D’Youville School of Pharmacy Portfolio Requirements

❑ Students must complete 2 co-curricular activities every semester.
○ Definition: Any activity that is done outside of school.
❑ Students must complete 1 pharmacy-related service (service learning) activity every semester.
○ Definition: Activity that is pharmacy related, has a pharmacy preceptor, and allows students to apply pharmacy skills.
❑ Students must have evidence of achieving the following professionalism/self-awareness activities by the end of the P1 year*.
○ Attending DYCSOP White Coat Ceremony (4.1).
○ Attend a chapter meeting of a professional student organization (4.1/4.4).
○ Complete APhA Career Pathways.
○ Complete certification in:
  ▪ APhA Immunization Delivery (4.1/4.4),
  ▪ Basic Life Support and CPR (4.1/4.4).
❑ Students must have evidence of achieving the following professionalism/self-awareness activities by the end of the P2 year*.
○ Obtaining a New York State Pharmacy Internship Permit (4.4).
○ Attend a chapter meeting of a professional student organization (4.1/4.4).
○ Attending one meeting of a local pharmacy organization/CE Meeting (4.1).
❑ Students must have evidence of achieving the following professionalism/self-awareness activities by the end of the P3 year*.
○ Attend a chapter meeting of a professional student organization (4.1/4.4).
○ Complete certification in:
  ▪ APhA Medication Therapy Management (4.1/4.4).
❑ Students must complete the following by the end of the P3 year:
○ Completion of 18 hours of pharmacy-related service (service learning),
○ Show evidence of achieving all eight co-curricular educational outcomes **at least once**,
○ Activities indicated by * above may not be utilized to fit this requirement.

## Appendix B. Focus Group Questions

Paper and ePortfolio
1. How many co-curricular and service-learning activities did you complete this semester? 2. In your own words, describe the purpose of co-curricular activities 3. How did the co-curricular requirements impact your choices of activities? 4. Which factors contribute to your selection of activities? 5. What do you like about the current process/framework? 6. How do you find out about activities? What would be the ideal method to communicate activities from your point of view? 7. How do you figure out mapping of activities? 8. What is still confusing in relation to ePortfolio requirements and how can we resolve confusion? 9. Do you find reflection difficult to write, what difficulties do you face when you write a reflection? 10. Do you prefer the mini-reflections or would you prefer one full-length reflection at the end of the semester or academic year? 11. What do you like about the current process/framework? Any suggestions for improvement? 12. If you would not have to write a reflection, how would you provide evidence that the participation in the activity contributed to your development? 13. Can you describe a typical meeting with your advisor where you discuss the co-curricular framework? 14. How can your advisor help you, from your perspective, in relation to the co-curricular framework and helping you track your progress?
ePortfolio Only
1. Is working on your ePortfolio a valuable activity? 2. Do you find it difficult to keep track of your activities in PharmAcademic? 3. Do you feel supported by your advisor? Why or why not?
